# Racial and Ethnic Differences in the Clinical Presentation of Celiac Disease in the United States: A Multi-institutional Retrospective Analysis

**DOI:** 10.1016/j.gastha.2026.100940

**Published:** 2026-03-24

**Authors:** Joy Zhao, John C. Lin, Jason C. Lin, Stephanie M. Moleski

**Affiliations:** 1 Division of Gastroenterology and Hepatology, Sidney Kimmel Medical College, Thomas Jefferson University, Philadelphia, Pennsylvania; 2 Perelman School of Medicine, University of Pennsylvania, Philadelphia, Pennsylvania; 3 Division of Gastroenterology, Hepatology, and Nutrition, McGovern Medical School, University of Texas Health Science Center, Houston, Texas

Celiac disease (CeD) is a chronic autoimmune disorder triggered by gluten ingestion, affecting approximately 1% of the general US population.^1^ While incidence has increased recently, likely due to greater awareness and improved diagnostic tools,^1^ substantial disparities remain in who gets screened, diagnosed, and treated.^2^ Prior studies have shown that CeD is more commonly diagnosed in non-Hispanic White individuals and women,^2^ although emerging evidence suggests that racial and ethnic minorities may present with different symptom profiles and remain underdiagnosed.^3^ Recent research has found that certain nutrient/micronutrient deficiencies were more common in racial and ethnic minorities.^4^

Despite clear clinical guidelines and well-established associations with autoimmune conditions, only a small proportion of eligible patients are ever screened for CeD.^5^^,^^6^ This gap is particularly pronounced among Black, Hispanic, and Asian populations.^6^^,^^7^ Given the broad spectrum of CeD manifestations and the potential for delayed or missed diagnoses in underrepresented groups, this study aimed to characterize racial and ethnic differences in the clinical presentation and comorbidities of patients with CeD using a large, multi-institutional electronic health record database.

This study was exempt per Institutional Review Board guidelines. We conducted a retrospective cohort study using the TriNetX Research Network, a global federated health research platform that provides access to deidentified electronic medical records from over 120 healthcare organizations, primarily based in the United States. TriNetX includes data on demographics, diagnoses (coded using the International Classification of Diseases, 10th Revision), procedures (Current Procedural Terminology codes), laboratory results (Logical Observation Identifiers Names and Codes), and medications (RxNorm), allowing for large-scale population-level analyses.

We queried TriNetX to identify patients with a recorded diagnosis of CeD using International Classification of Diseases, 10th Revision code K90.0. Patients were categorized into cohorts based on self-identified race and ethnicity: White, African American/Black, Hispanic, and Asian. Patients from other racial or ethnic backgrounds were excluded due to small sample size. When comparing respective cohorts, analysis for baseline characteristics on demographics, comorbidities, symptoms, and selected laboratory abnormalities were collected from the period preceding the index event, which was date of CeD diagnosis in the electronic medical record. Prior comorbidities, symptoms, and lab work of interest were selected based on what was commonly associated with CeD (Table 1).

**Table 1:** Basic Demographic Cohort Comparison of Patients With Celiac Disease

Characteristic	White	Black	Hispanic	Asian
Total number of patients in cohort	159,544	5627	3919	2357
Average age, in years	47	47	37	41
Female (%)	71.9%	74.0%	70.4%	67.4%

Descriptive analyses were conducted using the TriNetX analytics platform. Baseline characteristics were compared across cohorts using chi-square tests for categorical variables and t-tests for continuous variables. *P* < .05 was considered significant.

In total, 171,447 patients with CeD were included; 93% (n = 159,544) were White, 3% (n = 5627) were Black, 2% (n = 3919) were Hispanic, and 1% (n = 2357) were Asian. The mean age was 43 years old; an average of 70.9% of the patients was female as seen in Table 1.

The 10 most common characteristics of clinical presentation are presented in Table 2. Supplementary Table 1 shows the full comparison of different presentations. Black patients were more likely to have histories of constipation (30% vs 20%, *P* < .0001), adult failure to thrive (2% vs 1%, *P* < .0001), abnormal weight loss 13% vs 9%, *P* < .0001), recurrent seizure (5% vs 3%, *P* < .0001), and hypokalemia (13% vs 7%, *P* < .0001) compared to other races, specifically White patients with CeD. Black and Hispanic patients more commonly reported prior nausea and vomiting (33% vs 25%, *P* < .0001; 33% vs 25%, *P* < .0001) and skin paresthesia (12% vs 9%, *P* < .0001; 11% vs 9%, *P* = .0002), compared to White counterparts, respectively. Asian and Hispanic patients were more likely to have gaseous abdominal distention (16% vs 12%, *P* < .0001; 15% vs 12%, *P* < .0001) compared to White patients with CeD, respectively. White and Asian patients were more likely to have osteoporosis.

**Table 2:** Ten Most Common Characteristics of Clinical Presentation Among Adults With Celiac Disease

Ranking of traits	White (% occurrence)	Black (% occurrence)	Hispanic (% occurrence)	Asian (% occurrence)
1	Malaise and fatigue (29%)	Nausea and vomiting (33%)	Nausea and vomiting (33%)	Malaise and fatigue (27%)
2	Nausea and vomiting (25%)	Constipation (30%)	Constipation (27%)	Constipation (22%)
3	History of hypothyroidism (23%)	Malaise and fatigue (28%)	Malaise and fatigue (26%)	History of hypothyroidism (20%)
4	Constipation (20%)	Iron deficiency anemia (19%)	History of hypothyroidism (18%)	Nausea and vomiting (20%)
5	Abdominal distension, gaseous (12%)	History of hypothyroidism (17%)	Headache of unspecified etiology (17%)	Abdominal distension, gaseous (16%)
6	Headache of unspecified etiology (12%)	Headache of unspecified etiology (16%)	Abdominal distension, gaseous (15%)	Iron deficiency anemia (15%)
7	Iron deficiency anemia (11%)	Hypokalemia (13%)	Iron deficiency anemia (14%)	Headache of unspecified etiology (11%)
8	Abnormal weight loss (9%)	Abdominal distension, gaseous (13%)	Paresthesias of skin (11%)	Abnormal weight loss (9%)
9	Paresthesias of skin (9%)	Abnormal weight loss (13%)	Abnormal weight loss (9%)	Paresthesias of skin (9%)
10	Hypokalemia (7%)	Paresthesias of skin (12%)	Type 1 diabetes mellitus (8%)	Gas pain (6%)

In this large, retrospective cohort study using TriNetX data, we identified significant differences in the clinical presentation and comorbidities of CeD across racial and ethnic groups in the United States. Although CeD has traditionally been associated with non-Hispanic White populations,^2^^,^^3^ our findings reinforce the need for heightened clinical suspicion in historically underdiagnosed populations, including African American, Hispanic, and Asian individuals.

Our study supports prior findings that CeD remains underdiagnosed in non-White populations, despite comparable, if not more severe, symptomatology and burden of autoimmune disease.^3^^,^^4^ Structural factors, such as implicit bias, limited access to specialist care, and narrow diagnostic heuristics that anchor on European ancestry, may contribute to disparities in screening and diagnosis.^6^^,^^8^ The predominance of female patients across all cohorts is consistent with the known sex distribution of CeD and autoimmune conditions more broadly.^1^^,^^2^

This study highlights the urgent need to reconsider current diagnostic practices and guidelines, especially given that CeD symptoms may manifest differently based on racial or ethnic background. Increased awareness and equitable application of screening protocols are essential. Furthermore, the development of tailored risk-assessment tools that incorporate diverse symptom patterns could improve early detection in underserved populations.

Study limitations include reliance on retrospective electronic health record data, which may be subject to coding inaccuracies or incomplete documentation. Additionally, race and ethnicity were self-reported and may not fully capture the complexity of sociocultural factors influencing disease presentation. Some relevant data, including dermatitis herpetiformis and vitamin deficiencies, were underreported and may limit interpretability. Finally, the timing from symptom to diagnosis is unable to be parsed out given the structure of the TriNetX database, where individual patient information cannot be accessed on a granular level. Nevertheless, this study provides one of the largest contemporary assessments of CeD presentations across racial and ethnic lines and underscores the need for more inclusive diagnostic frameworks.

While White patients remain the majority of diagnosed CeD cases, non-White patients exhibit distinct and often more severe clinical profiles. These differences underscore the need for broader diagnostic consideration and culturally sensitive approaches to CeD screening. Future research should explore the interplay of social determinants of health, healthcare access, and genetic predispositions to optimize equitable diagnosis and care in CeD.

## Supplementary Materials

Supplementary Table 1

Extended PDF
