# Supplementary material for: Racial and Ethnic Differences in the Clinical Presentation of Celiac Disease in the United States: A Multi-institutional Retrospective Analysis
Source: Gastro Hep Adv. 2026 Mar 24;5(6):100940. doi: 10.1016/j.gastha.2026.100940 (PMC13141494; doi:10.1016/j.gastha.2026.100940)
Supplement: Supplementary Table 1 [file mmc1.pdf]

Supplementary Table 1. Full Comparison of Clinical Presentation Between Celiac Cohorts by Race and Ethnicity

**Characteristic** (*P-Value*[a], % Comparison[b])	**White vs. African American**	**White vs. Hispanic**	**White vs. Asian**	**African American vs. Hispanic**	**African American vs. Asian**	**Hispanic vs. Asian**
Type 1 diabetes mellitus	$P < .0001$ 6% vs 9%	$P = .0001$ 6% vs. 8%	$P = .011$ 6% vs. 5%	$P = .089$ 9% vs 8%	$P < .0001$ 9% vs 5%	$P < .0001$ 8% vs 5%
Malaise and fatigue	$P = .333$ 29% vs 28%	$P < .0001$ 29% vs. 26%	$P = .015$ 29% vs. 27%	$P < .0001$ 28% vs 24%	$P = .100$ 28% vs 26%	$P = .391$ 26% vs 27%
Abdominal distension (gaseous)	$P = .210$ 12% vs 13%	$P < .0001$ 12% vs. 15%	$P < .0001$ 12% vs. 16%	$P = .024$ 13% vs 14%	$P = .002$ 13% vs 15%	$P = .618$ 15% vs 16%
Gas pain	$P = .112$ 5% vs 5%	$P = .004$ 5% vs. 6%	$P < .0001$ 5% vs. 6%	$P = .698$ 5% vs 5%	$P = .027$ 5% vs 6%	$P = .238$ 6% vs 6%
Nausea and vomiting	$P < .0001$ 25% vs 33%	$P < .0001$ 25% vs. 33%	$P < .0001$ 25% vs. 20%	$P = .471$ 33% vs 32%	$P < .0001$ 33% vs 20%	$P < .0001$ 33% vs 20%
Constipation	$P < .0001$ 20% vs 30%	$P < .0001$ 20% vs. 27%	$P = .141$ 20% vs. 22%	$P = .004$ 29% vs 27%	$P < .0001$ 29% vs 22%	$P < .0001$ 27% vs 22%
Failure to thrive (child)	$P < .0001$ 2% vs 3%	$P < .0001$ 2% vs. 4%	$P < .0001$ 2% vs. 5%	$P = .001$ 3% vs 4%	$P < .0001$ 3% vs 5%	$P = .124$ 4% vs 5%
Adult failure to thrive	$P < .0001$ 1% vs 2%	$P = .083$ 1% vs. 1%	$P = .241$ 1% vs. 1%	$P < .0001$ 2% vs 1%	$P = .0002$ 2% vs 1%	$P = .872$ 1% vs 1%
Iron deficiency	$P = .148$ 5% vs 5%	$P = .194$ 5% vs. 5%	$P = .013$ 5% vs. 6%	$P = .637$ 5% vs 5%	$P = .220$ 5% vs 6%	$P = .271$ 5% vs 6%
Iron deficiency anemia, unspecified	$P < .0001$ 11% vs 19%	$P < .0001$ 11% vs. 14%	$P < .0001$ 11% vs. 15%	$P < .0001$ 19% vs 14%	$P < .0001$ 19% vs 15%	N/A
Headache, unspecified	$P < .0001$ 12% vs 16%	$P < .0001$ 12% vs. 17%	$P =.160$ 12% vs. 11%	$P =.165$ 16% vs 17%	$P < .0001$ 16% vs 11%	$P < .0001$ 17% vs 11%
Abnormal weight loss	$P < .0001$ 9% vs 13%	$P = .815$ 9% vs. 9%	$P = .905$ 9% vs. 9%	$P < .0001$ 13% vs 9%	$P < .0001$ 13% vs 9%	$P = .810$ 9% vs 9%

Preterm labor	*P* = .025<br>1% vs 1%	*P* = .037<br>1% vs. 1%	*P* = .620<br>1% vs. 1%	*P* = .995<br>1% vs 1%	*P* = .536<br>1% vs 1%	*P* = .422<br>1% vs 1%
Single stillbirth	*P* < .0001<br>0% vs 0%	*P* = .172<br>0% vs 0%	*P* < .0001<br>0% vs. 0%	*P* = .010<br>0% vs 0%	*P* = .041<br>0% vs 0%	*P* < .0001<br>0% vs 0%
Twins, both stillborn	*P* < .0001<br>0% vs 0%	*P* = .620<br>0% vs. 0%	*P* = .701<br>0% vs. 0%	*P* = .010<br>0% vs 0%	*P* = .042<br>0% vs 0%	N/A
Other multiple births, all stillborn	*P* = .553<br>0% vs 0%	*P* = .620<br>0% vs. 0%	*P* = .701<br>0% vs. 0%	N/A	N/A	N/A
Other osteoporosis without current pathological fracture	*P* = .003<br>2% vs 1%	*P* = .002<br>2% vs. 1%	*P* = .093<br>2% vs. 2%	*P* = .279<br>1% vs 1%	*P* = .002<br>1% vs 2%	*P* = .001<br>1% vs 2%
Paresthesia of skin	*P* < .0001<br>9% vs 12%	*P* = .0002<br>9% vs. 11%	*P* = .403<br>9% vs. 9%	*P* = .240<br>12% vs 11%	*P* = .0003<br>12% vs 9%	*P* = .004<br>11% vs 9%
Epilepsy and recurrent seizures	*P* < .0001<br>3% vs 5%	*P* = .524<br>3% vs. 3%	*P* = .171<br>3% vs. 2%	*P* = .001<br>5% vs 3%	*P* < .0001<br>5% vs 2%	*P* = .133<br>3% vs 2%
Vitiligo	*P* < .0001<br>0% vs 1%	*P* = .001<br>0% vs. 1%	*P* = .001<br>0% vs. 1%	*P* = .600<br>1% vs 1%	*P* = .976<br>1% vs 1%	*P* = .721<br>1% vs 1%
Rheumatoid arthritis, unspecified	*P* < .0001<br>2% vs 3%	*P* = .001<br>2% vs. 3%	*P* = .073<br>2% vs. 2%	*P* = .836<br>3% vs 3%	*P* = .001<br>3% vs 2%	*P* = .001<br>3% vs 2%
Psoriasis	*P* < .0001<br>3% vs 2%	*P* = .007<br>3% vs. 2%	*P* = .042<br>3% vs. 2%	*P* = .017<br>2% vs 2%	*P* = .060<br>2% vs 2%	*P* = .943<br>2% vs 2%
Systemic lupus erythematosus (SLE)	*P* < .0001<br>2% vs 3%	*P* = .006<br>2% vs. 2%	*P* = .963<br>2% vs. 2%	*P* = .001<br>3% vs 2%	*P* < .0001<br>3% vs 2%	*P* = .1090<br>2% vs 2%
Other hypothyroidism	*P* < .0001<br>20% vs 13%	*P* < .0001<br>20% vs. 15%	*P* < .0001<br>20% vs. 17%	*P* = .007<br>13% vs 15%	*P* < .0001<br>13% vs 16%	*P* = .148<br>15% vs 17%

Thyrotoxicosis, hyperthyroidism	*P* < .0001<br>3% vs 4%	*P* = .925<br>3% vs. 3%	*P* = .383<br>3% vs. 3%	*P* = .012<br>4% vs 3%	*P* = .006<br>4% vs 3%	*P* = .516<br>3% vs 3%
Down syndrome	*P* = .266<br>1% vs 1%	*P* < .0001<br>1% vs. 2%	*P* = .159<br>1% vs. 1%	*P* = .038<br>1% vs 2%	*P* = .567<br>1% vs 1%	*P* = .222<br>2% vs 1%
Hypocalcemia	*P* < .0001<br>2% vs 3%	*P* = .485<br>2% vs. 2%	*P* = .463<br>2% vs. 2%	*P* = .109<br>3% vs 2%	*P* = .159<br>3% vs 2%	*P* = .886<br>2% vs 2%
Hypokalemia	*P* < .0001<br>7% vs 13%	*P* = .335<br>7% vs. 7%	*P* = .001<br>7% vs. 5%	*P* < .0001<br>13% vs 7%	*P* < .0001<br>13% vs 5%	*P* = .001<br>7% vs 5%
Acute metabolic acidosis	*P* = .004<br>0% vs 0%	*P* < .0001<br>0% vs. 0%	*P* < .0001<br>0% vs. 0%	*P* = .350<br>0% vs 0%	*P* = .041<br>0% vs 0%	*P* = .250<br>0% vs 0%
Chronic metabolic acidosis	*P* < .0001<br>0% vs 0%	*P* = .334<br>0% vs. 0%	*P* = .454<br>0% vs. 0%	*P* = .010<br>0% vs 0%	*P* = .042<br>0% vs 0%	N/A
Folate deficiency anemia	*P* < .0001<br>0% vs. 1%	*P* = .825<br>0% vs. 0%	*P* = .168<br>0% vs. 0%	*P* = .034<br>1% vs 0%	*P* = .437<br>1% vs 0%	*P* = .250<br>0% vs 0%

[a] $P < .05$ is considered significant
[b]Percentage for respective cohorts
